# Draft Genome Sequence of Lysinibacillus sp. Strain A1, Isolated from Malaysian Tropical Soil

**DOI:** 10.1128/genomeA.00095-15

**Published:** 2015-03-26

**Authors:** Kok-Gan Chan, Jian Woon Chen, Chien-Yi Chang, Wai-Fong Yin, Xin-Yue Chan

**Affiliations:** a Division of Genetics and Molecular Biology, Institute of Biological Sciences, Faculty of Science, University of Malaya, Kuala Lumpur, Malaysia; b Interdisciplinary Computing and Complex BioSystems (ICOS) Research Group, School of Computing Science, Newcastle University, Newcastle upon Tyne, United Kingdom; c Centre for Bacterial Cell Biology, Medical School, Newcastle University, Newcastle upon Tyne, United Kingdom

## Abstract

In this work, we describe the genome of Lysinibacillus sp. strain A1, which was isolated from tropical soil. Analysis of its genome sequence shows the presence of a gene encoding for a putative peptidase responsible for nitrogen compounds.

## GENOME ANNOUNCEMENT

Lysinibacillus spp. are Gram-positive rod bacteria that can survive in a wide range of extreme environments, including heavy metal-contaminated soil (1–5). Even though Lysinibacillus sp. is well known for its bioremediation potential, knowledge about its biodegradation ability is limited. In this study, we analyzed the bacterial genome of Lysinibacillus sp. strain A1. In addition, we determined the peptidase-coding gene from its whole-genome sequence.

Lysinibacillus sp. strain A1 was isolated from soil surface (Rimba Ilmu, Kuala Lumpur, Malaysia) using KGm medium and cultivated in Luria-Bertani medium (6, 7). The genomic DNA was isolated using a MasterPure DNA purification kit (Epicenter, USA) (8). The extracted DNA was quantified and qualified using Qubit version 2.0 (Invitrogen, USA) and Nanodrop (Thermo Scientific, USA) (9). The high-quality DNA was sent for next-generation sequencing (NGS) library preparation using a Nextera DNA sample preparation kit (Illumina, USA) and sequenced using a MiSeq 600-cycle sequencing kit (version 3) on a MiSeq platform (Illumina, USA) (9). The preliminary analysis was performed with CLC Genomic Workbench version 7.5, while the annotation are performed using the NCBI prokaryote genome annotation pipeline (version 2.9) and NCBI BLAST against the nr database (10, 11).

The NGS of the Lysinibacillus sp. strain A1 genome by MiSeq generated 1.2 million paired-end reads. These reads were trimmed and assembled into 57 contigs with average coverage of 42-fold and an *N*_50_ of 206,495 bp. The genome size is 4.75 Mbps with 37.45% G+C content. This genome carried 4,449 coding DNA sequences (CDS) and coded for 4,693 genes and 159 pseudogenes.

A peptidase-coding gene was detected in contig 1 in the draft genome of Lysinibacillus sp. strain A1. The length of this gene is 1,089 bp, and it is located at a position between 182,713 and 183,801 bp at contig 1. Based on the gene alignment and comparison, it is classified into peptidase M28. It has been reported that peptidase is used by both macro- and microorganisms to break down protein by hydrolyzing its peptide bond in order to acquire nutrients (12). The cocatalytic active site of peptidase M28 was formed by two zinc ions (13, 14). To our knowledge, the production of peptidase M28 by Lysinibacillus has not been reported. Thus, our work on the genome and peptidase gene of Lysinibacillus sp. strain A1 will lead to further understanding on the function of this protein hydrolysis enzyme.

### Nucleotide sequence accession numbers.

This draft genome was deposited into DDBJ/EMBL/GenBank under the accession number JSZM00000000. The version described in this paper is the first version, JSZM01000000.

## References

[1] Kong D, Wang Y, Zhao B, Li Y, Song J, Zhai Y, Zhang C, Wang H, Chen X, Zhao B, Ruan Z 2014 *Lysinibacillus halotolerans* sp. nov., isolated from saline-alkaline soil. Int J Syst Evol Microbiol 64:2593–2598. doi:10.1099/ijs.0.061465-0. 24814335

[2] He M, Li X, Liu H, Miller SJ, Wang G, Rensing C 2011 Characterization and genomic analysis of a highly chromate resistant and reducing bacterial strain *Lysinibacillus fusiformis* ZC1. J Hazard Mater 185:682–688. doi:10.1016/j.jhazmat.2010.09.072. 20952126

[3] Miwa H, Ahmed I, Yokota A, Fujiwara T 2009 *Lysinibacillus parviboronicapiens* sp. nov., a low-boron-containing bacterium isolated from soil. Int J Syst Evol Microbiol 59:1427–1432. doi:10.1099/ijs.0.65455-0. 19502328

[4] Rahman A, Nahar N, Nawani NN, Jass J, Desale P, Kapadnis BP, Hossain K, Saha AK, Ghosh S, Olsson B, Mandal A 2014 Isolation and characterization of a *Lysinibacillus* strain B1-CDA showing potential for bioremediation of arsenics from contaminated water. J Environ Sci Health A Tox Hazard Subst Environ Eng 49:1349–1360. doi:10.1080/10934529.2014.928247. 25072766

[5] Kumar M, Yadav AN, Tiwari R, Prasanna R, Saxena AK 2014 Deciphering the diversity of culturable thermotolerant bacteria from Manikaran hot springs. Ann Microbiol 64:741–751. doi:10.1007/s13213-013-0709-7.

[6] Chen JW, Koh C-L, Sam C-K, Yin W-F, Chan K-G 2013 Short chain *N*-acyl homoserine lactone production by soil isolate *Burkholderia* sp. strain A9. Sensors 13:13217–13227. doi:10.3390/s131013217. 24084115 PMC3859060

[7] Chan K-G, Yin W-F, Sam C-K, Koh C-L 2009 A novel medium for the isolation of *N*-acylhomoserine lactone-degrading bacteria. J Ind Microbiol Biotechnol 36:247–251. doi:10.1007/s10295-008-0491-x. 18946694

[8] Han-Jen RE, Wai-Fong Y, Kok-Gan C 2013 *Pandoraea* sp. RB-44, a novel quorum sensing soil bacterium. Sensors 13:14121–14132. doi:10.3390/s131014121. 24145919 PMC3859112

[9] Chan X-Y, Chua KH, Yin W-F, Puthucheary SD, Chan K-G 2014 Whole-genome analysis of *Aeromonas hydrophila* strain 187, exhibiting quorum-sensing activity. Genome Announc 2(6):e01360-01314. doi:10.1128/genomeA.01360-14. 25540357 PMC4276835

[10] NCBI Resource Coordinators 2014. Database resources of the national center for biotechnology information. Nucleic Acids Res 42:D7–D17. doi:10.1093/nar/gkt1146. PMC3965057 24259429

[11] McCulloch JA, de Oliveira VM, de Almeida Pina AV, Pérez-Chaparro PJ, de Almeida LM, de Vasconcelos JM, de Oliveira LF, da Silva DE, Rogez HL, Cretenet M, Mamizuka EM, Nunes MR 2014 Complete genome sequence of *Lactococcus lactis* strain ai06, an endophyte of the Amazonian açaí palm. Genome Announc 2:e01225-01214. doi:10.1128/genomeA.01225-14. 25414513 PMC4239368

[12] Kooi C, Sokol PA 1996 Differentiation of thermolysins and serralysins by monoclonal antibodies. J Med Microbiol 45:219–225. doi:10.1099/00222615-45-3-219. 8810950

[13] Tsukamoto T, Flanary JM, Rojas C, Slusher BS, Valiaeva N, Coward JK 2002 Phosphonate and phosphinate analogues of *N*-acylated γ- glutamylglutamate: potent inhibitors of glutamate carboxypeptidase II. Bioorg Med Chem Lett 12:2189–2192. doi:10.1016/S0960-894X(02)00360-8. 12127534

[14] Fundoiano-Hershcovitz Y, Rabinovitch L, Langut Y, Reiland V, Shoham G, Shoham Y 2004 Identification of the catalytic residues in the double-zinc aminopeptidase from *Streptomyces griseus*. FEBS Lett 571:192–196. doi:10.1016/j.febslet.2004.07.001. 15280041

